# Presentation of a case of Bruton type primary agammaglobulinemia in Guinea

**DOI:** 10.11604/pamj.2020.36.385.24771

**Published:** 2020-08-31

**Authors:** Kaba Condé, Hugues Ghislain Atakla, Mamadou Ciré Barry, Mohamed Lamine Condé, Malé Doré

**Affiliations:** 1 Rheumatology Department, Ignace Deen University Hospital Center, Conakry, Guinea,; 2 Neurology Department, Ignace Deen University Hospital Center, Conakry, Guinea,; 3 Pediatric Department, Ignace Deen University Hospital Center, Conakry, Guinea

**Keywords:** Agammaglobulinemia, immunoglobulins, Burton’s disease, Guinea

## Abstract

X-linked agammaglobulinemia (XLA) is a rare genetic disease caused by a mutation in the Bruton tyrosine kinase (BTK) gene. It is characterized by a profound deficiency of B cells and a decrease in all classes of immunoglobulins (Ig). We report one case in a 3-year-old boy seen for recurrent acute otitis media, perineal abscess, oligoarthritis. The serum immunoglobulin (Ig) assay showed an IgG level of 0.6g/l. IgM and IgA are indosable. Marrow immunophenotyping showed an absence of precursor B less than 1%. Molecular biology confirmed Burton's disease (stop mutation, C37C) in exon 2 of the BTK gene. Treatment with intravenous immunoglogulin was started.

## Introduction

X-linked agammaglobulinemia (XLA) is a rare genetic disease caused by a mutation in the Bruton tyrosine kinase (BTK) gene [1]. XLA is characterized by a profound deficiency of B cells and a decrease in all classes of immunoglobulins (Ig) [2]. Patients with XLA have markedly reduced, weak or absent circulating B cells [3]. XLA patients suffer from recurrent sino-pulmonary infections such as otitis media, sinusitis, bronchitis, pneumonia and gastrointestinal infections. Joint involvement is rare [4,5]. We report one case of primary agammaglobulinemia of the Bruton type with joint involvement.

## Patient and observation

**Figure 1:**
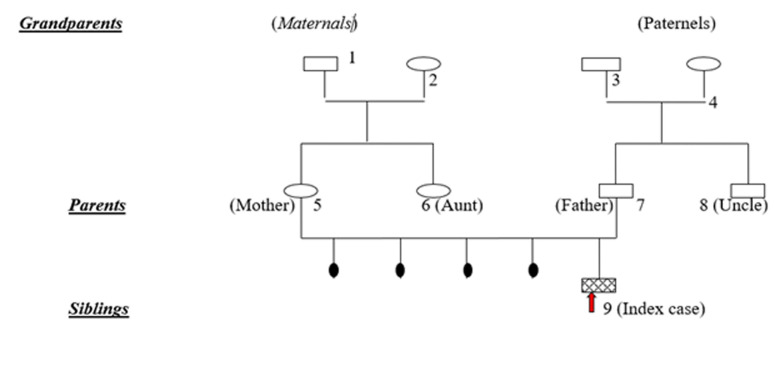
the family tree (1): non-insulin dependent diabetes (2): iron deficiency anemia (3): MTA (4): renal MTA (5): HbAS heterozygous (6): myopia (9): index case (agammaglobulinemia probably related to X)

It was a 3-year-old boy, the first child of a non-inbred couple (Figure 1), born of a natural pregnancy that was carried to term (39 weeks and 4 days). The neonatal period was uneventful and his vaccination is up to date. He was apparently well until the age of 6 months when he was admitted to the emergency room with fever secondary to recurrent acute otitis media. In addition, he suffered from perineal abscess, recurrent upper and lower respiratory tract infection. At the age of one year, there were two episodes of pneumonia requiring hospitalization and intravenous antibiotic therapy. Two years later, he had pain and swelling in his joints at the wrists and knees with an inflammatory appearance. On examination, he weighed 11kg and measured 81cm. He is in perfect general condition. There was pain and swelling of the wrists and knees. The tonsils were very small and there were no palpable ganglions. The cardio-pulmonary, abdominal and neurological examination was unremarkable. The biological work-up had shown a haemoglobin level of 11.7g/dl, white blood cells at 5390/mm^3^ with a formula showing deep and persistent neutropenia at 500mm^3^. The sedimentation rate (SV) was 12mm at the first hour as the C-reactive protein was 8mg/l. Renal and hepatic function was normal. Antinuclear antibodies and rheumatoid factor were negative. X-rays of hands/wrists and knees were normal. In view of the recurrent infections, a screening for immune deficiency was performed. The serum immunoglobulin assay showed an IgG level of 0.6g/l (age standard is 2-6g/l). IgM and IgA were indistinguishable. Spinal cord puncture revealed a rich marrow with a 10% blast rate and the absence of mature neutrophils in the marrow with a myeloid maturation blockage. No morphological abnormalities. Marrow immuno-phenotyping showed an absence of precursor B (less than 1% CD19, CD20, CD22, CD24 lymphocytes). A lymphocytosis B, absence of IgG and normal cellular immunity, age and sex support the diagnosis of Bruton's disease. Whether it is most likely remains to be confirmed by molecular biology (BTK mutation). Treatment with intravenous immunoglobulin every four weeks has been started. Molecular biology being impossible to perform in Guinea, after parental consent, a DNA sample was taken and sent to Holland to search for a mutation of the BTK gene located in Xq21.3-q22. After several months, we had genetic confirmation of Bruton's disease (stop mutation, C37C) in exon 2 of the BTK gene. The patient responded well to intravenous Ig, no more infectious problems, no more joint swelling.

## Discussion

X-linked agammaglobulinemia is a rare genetic disease, first described in 1952 by Colonel Ogden Bruton [6]. Its incidence is estimated at 1/190,000 male births or 1/379,000 live births [7]. In black Africa, few data are available. The delay in diagnosis is considerable and many children die before diagnosis [4]. We report the case of a three-year-old boy. XLA is caused by a mutation in the Bruton tyrosine kinase (BTK) gene, located on the long arm of the X chromosome. BtKK is involved in the maturation of pre-B cells into mature B cells [8]. After mutation of the BTK gene, there is a failure of B cell development and affected patients have a significantly low level (<1%) of mature B cells in peripheral blood [4,8]. They fail to generate plasma cells and therefore have markedly low levels of all IgG classes with virtually no humoral response [3,9]. It also leads to a reduction in the size of lymph nodes and tonsils, as reported in our case. Most of the mutations in the BTK gene are familial, the mothers of the affected individuals being healthy carriers [4,10]. About 50% of patients have a family history of a previously affected family member [7]. However, due to technical and financial constraints, we were unable to perform genetic testing on other family members. Children with XLA become symptomatic between 6 and 12 months, once the passively transferred protective maternal IgG fades [4]. Indeed, the first symptoms in our patient occurred at the age of 6 months. Most patients have recurrent respiratory, ENT and gastrointestinal tract infections [4,11]. However, our patient suffered from an uncommon manifestation in XLA patients, arthritis. Approximately 10-30% of patients with XLA have arthritis and it is usually mono- or oligoarthritis of the large joints [11,12]. There is currently no cure for patients with XLA. The defective gene cannot be repaired or replaced [13]. However, the antibodies are provided in the form of Ig antibodies that can be administered intravenously or subcutaneously [4,13]. Our patient is treated with intravenous Ig every four weeks.

## Conclusion

In black Africa, the delay in diagnosis is considerable and many children die before diagnosis. Diagnosis and treatment are expensive, beyond the reach of the average household. An effort by doctors and the government is needed to improve the prognosis of these children.
